# Abu Zayd Ahmed ibn Sahl Al-Balkhi (850-934): A Pioneer in the Field of Psychotherapy and Mental Health

**DOI:** 10.7759/cureus.67998

**Published:** 2024-08-28

**Authors:** Sarhang I Saeed, Jaafar O Ahmed, Karwan Kakamad, Zana Najmadden

**Affiliations:** 1 Clinical Psychology, Koya University, Koya, IRQ; 2 Psychology, Soran University, Soran, IRQ; 3 Scientific Research, Kscien Organization, Sulaimanyah, IRQ; 4 Research, University of Halabja, Halabja, IRQ

**Keywords:** psychosomatic, islamic psychology, al-balkhi, cognitive-behavioral therapy (cbt), psychotherapy, mental health, historical vignette

## Abstract

Abu Zayd Ahmed ibn Sahl Al-Balkhi (850-934) was a versatile scholar during the Islamic Golden Age, who made significant contributions in mental health, psychology, medicine, geography, and philosophy. Al-Balkhi delved into the intricate connection between psychological and physical health, contributing to the early understanding of psychosomatic medicine. His innovative understanding of mental health conditions such as anxiety and depression established the foundation for the subsequent development of cognitive-behavioral therapy (CBT). Al-Balkhi's multidisciplinary approach, shaped by the guidance of his instructor Al-Kindi and the intellectually vibrant atmosphere of Baghdad, enabled him to assimilate knowledge from other traditions. His enduring legacy is acknowledged for its tremendous influence on historical and contemporary scientific thinking, establishing his position as a trailblazing figure in mental health and psychology. This narrative review combines historical and contemporary views to assess Al-Balkhi's lasting legacy in the field of psychotherapy and mental health.

## Introduction and background

Abu Zayd Ahmed ibn Sahl Al-Balkhi (850-934) was a highly accomplished scholar during the Islamic Golden Age. He made significant contributions to various disciplines, such as medicine, psychology, mathematics, and geography [1,2]. Al-Balkhi, born in Shamistiyan, a village near Balkh in what is now Afghanistan, had his education in Baghdad, a prominent center of intellectual activity during that era [3]. He received education from the renowned philosopher Al-Kindi, greatly impacting his interdisciplinary knowledge approach [4,5].

Al-Balkhi is mostly recognized for his groundbreaking contributions to the realm of psychology and mental well-being. Regarded as one of the earliest comprehensive researchers on psychosomatic medicine, his influential work "Masalih al-Abdan wa al-Anfus" (Sustenance for Bodies and Souls) stands as a key text [1]. In this book, he examines the interaction between physical and mental well-being, promoting comprehensive approaches to health long before these ideas became popular in Western medicine [6]. Al-Balkhi's observations were revolutionary, including his comprehension that mental health problems might arise from both psychological and physiological sources, and his acknowledgment of the significance of emotional well-being in general health [7].

In addition, Al-Balkhi's work encompassed the fields of geography and cartography. He played a crucial role in the Al-Balkhi school of terrestrial mapping, making substantial contributions to the advancement of Islamic geography [2]. His methodical approach to map-making and his focus on empirical observation was revolutionary during his era, establishing the foundation for future progress in the area [8]. Al-Balkhi's many contributions epitomize the abundant intellectual heritage of the Islamic Golden Age and underscore his pivotal role as a vital connection between ancient and contemporary scientific thinking [9]. His legacy is being honored in other fields, highlighting his reputation as a trailblazer in holistic and integrative approaches to knowledge [8]. The aim of this narrative review is to analyze the contributions of Al-Balkhi to the fields of mental health, psychotherapy, and psychosomatic medicine.

## Review

Al-Balkhi’s life and career

Abu Zayd Ahmed ibn Sahl Al-Balkhi, born in Shamistiyan in the Province of Balkh, Greater Khorasan (present-day Afghanistan), was a distinguished intellectual during the years 850 to 934 CE [1]. He obtained his primary education in his village before relocating to Baghdad, which served as the preeminent hub of knowledge throughout the Abbasid Caliphate [10]. While in Baghdad, Al-Balkhi received an education under the renowned philosopher Al-Kindi, which greatly influenced his academic endeavors [7,11]. Al-Balkhi's career was characterized by his diverse and impactful contributions across multiple disciplines. Known for his innovative approach to combining real-world observations with organized geographic documentation, he was a prominent geographer who played a key role in the development of the Al-Balkhi school of terrestrial mapping [12]. His pioneering cartographic and geographical methodology significantly contributed to the advancement of knowledge regarding the physical arrangement of the world in medieval Islamic civilization [8].

**Figure 1:**
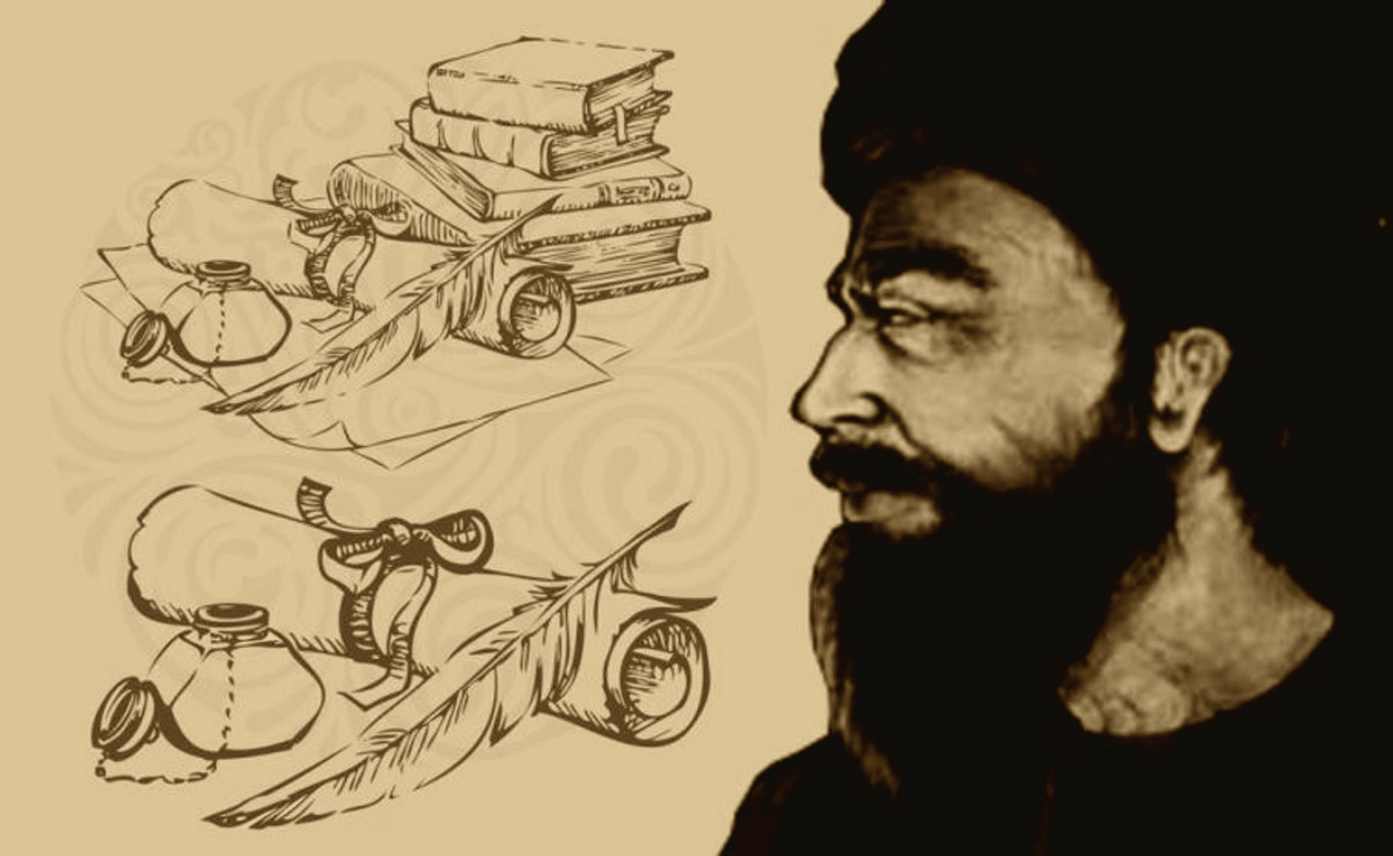
A portrait of Al-Balkhi (850-934) Reproduced with permission from About Islam [14].

Al-Balkhi made significant contributions to the medical field by writing the pioneering book "Masalih al-Abdan wa al-Anfus" (Sustenance for Bodies and Souls). This literature is an early and thorough treatise that discusses both physical and mental health, promoting a holistic approach to well-being [2]. Al-Balkhi delineated and elucidated diverse mental health disorders and their corresponding symptoms, while also advocating for therapeutic approaches that underscore the significance of mental well-being in overall physical health [4,8]. He was a very important teacher whose views had a profound and lasting impact on both Islamic and Western medical traditions, his comprehensive approach to health and his pioneering approaches to geography highlight his status as a polymath who successfully connected many areas of knowledge [13]. Al-Balkhi's contributions established fundamental principles that paved the way for future progress in the fields of science and medicine, underscoring his lasting impact as a distinguished scholar (Figure 1) [4].

Influencers of Al-Balkhi

A wide range of scientists greatly influenced Al-Balkhi. His tutor, Al-Kindi, who was widely recognized as the "Philosopher of the Arabs," had a profound impact on his work [11]. Al-Kindi's incorporation of Greek philosophy, specifically the ideas of Aristotle and Plato, into Islamic intellectual traditions had a long-lasting influence on Al-Balkhi [8]. Al-Balkhi's comprehensive approach to science and medicine is influenced by his recognition of the significance of both physical and mental well-being [13,15].

The translation activity in Baghdad, which translated the works of Greek, Persian, and Indian intellectuals into Arabic, established a vast pool of knowledge from which Al-Balkhi could draw [7]. The amalgamation of these varied intellectual traditions into a unified framework of comprehension is evidence of the syncretic character of Islamic study throughout this era [2]. He lived in the same period as other renowned thinkers like Al-Razi and Al-Farabi, and, like them, he had a profound understanding of the extensive heritage of Islamic science and philosophy [16].

Al-Balkhi's work emerged from a dynamic intellectual environment, strongly shaped by his tutor Al-Kindi, the medical practices of ancient Greece, and the diverse scholarship of the Islamic Golden Age [8]. These influences empowered him to spearhead notable breakthroughs in the disciplines of geography, medicine, and psychology [17].

Books and publications of Al-Balkhi

Al-Balkhi was a highly productive writer who covered a wide range of subjects, including geography, medicine, and psychology [12]. The most famous piece of his repertoire is "Masalih al-Abdan wa al-Anfus" (Sustenance for Bodies and Souls). This literature represents one of the earliest extensive treatises on psychosomatic medicine, in which Al-Balkhi explores mental health conditions such as anxiety and depression, elucidating their symptoms and proposing therapeutic modalities [4]. Al-Balkhi not only made notable contributions to medicine but also achieved great improvements in geography [12].

Al-Balkhi's vast repertoire of writings highlights his significance as a prominent scholarly personality during the Islamic Golden Age, whose contributions are still acknowledged for their profoundness and impact [9]. His book "Kitab al-Bad' wa al-Tarikh" (The Book of Creation and History) is a prominent illustration of his contributions in this area. The original text's impact is acknowledged through allusions in subsequent works; despite its loss, this publication contributed to the wider academic endeavor of recording and comprehending the world using organized observations and modeling [8].

Balkhi's contributions also encompassed Islamic theology and philosophy. The author's writings demonstrated a fusion of several intellectual traditions, integrating components from Greek, Persian, and Indian origins [9]. This interdisciplinary approach not only propelled knowledge in his period but also established the foundation for future advancements in various domains [13]. He wrote over 60 books and manuscripts, thoroughly exploring various fields including geography, medicine, theology, politics, philosophy, poetry, literature, Arabic grammar, astrology, astronomy, mathematics, biography, ethics, sociology, and more [2,8,11]. Despite his expertise in various fields, his reputation as a great scholar primarily stemmed from his groundbreaking work in geography, which established him as the founder of the "Balkhi School" of terrestrial mapping [8]. Like other Muslim medieval scholars, unfortunately, many of these important manuscripts have either been lost or remain hidden in museums or inaccessible libraries [2,11,16]. Table 1 shows the most-known works of Abu Zayd Ahmed ibn Sahl Balkhi.

**Table 1:** Example of Al-Balkhi’s books This table is adapted from various scholarly sources [4,11,8,9].

Arabic name	English name	Description
Masalih al-Abdan wa al-Anfus	Sustenance for Bodies and Souls	A comprehensive discourse on the intricate relationship between physical and mental well-being.
Kitab al-Bad' wa al-Tarikh	The Book of Creation and History	Work on geography and history.
Kitab Naẓm al-Qur’ān	Quranic Systems	This work is known for its early and insightful analysis of the structure and coherence of the Qur'anic text.
Suwar al-Aqalim	Portrait of Territories	It is a geographical work that includes detailed maps and descriptions of various regions (climes) of the world known to the Islamic world at the time.

Works in the field of psychiatry and mental health

Al-Balkhi made noteworthy advancements in the realm of medicine, specifically through his innovative research on psychosomatic medicine [1,3,8]. One of his most significant medical works is "Masalih al-Abdan wa al-Anfus". This pioneering study showcases Al-Balkhi's revolutionary methodology in comprehending and addressing diverse medical ailments by taking into account both the physiological and psychological dimensions [6,12,18]. This book provides a thorough examination of many mental health issues, including anxiety, depression, and obsessive-compulsive disorders. He was one of the pioneers in identifying and providing detailed descriptions of these diseases, acknowledging their influence on overall health [2,7,13]. Al-Balkhi underscored the significance of promptly identifying and addressing medical conditions, pushing for a comprehensive healthcare approach that incorporates both mental and physical health methods [4,6]. His understanding of mental health was groundbreaking during his age and is now seen as a predecessor to contemporary cognitive-behavioral therapy (CBT) [13,19].

Al-Balkhi's work also encompassed the pragmatic aspects of psychological medicine. He offered comprehensive explanations of symptoms and suggested remedies for different illnesses, blending practical observations with theoretical understanding [7-9]. His focus on the interaction between the body and mind resulted in the creation of therapy approaches that targeted both physical symptoms and underlying psychological problems [12,20]. This dual emphasis represented a notable deviation from the prevalent medical norms of his time, which frequently regarded physical diseases and mental well-being as distinct things [3,4]. His radical concepts and thorough approaches established the basis for future progress in psychosomatic medicine and the therapy of mental health [7,21,22].

Al-Balkhi described several mental health illnesses, including anxiety (al-jaza), depression (al-huzn), obsessive-compulsive disorders (OCD), panic episodes (al-faza), and phobia (al-khawf) [2,20,23]. His book “Maṣāliḥ al-abdān wa-al anfus” likely made a clear differentiation between neuroses and psychosis and was the first to categorize neurotic disorders nearly a thousand years before Freud. He also provided detailed guidance on employing both rational and spiritual cognitive therapies to treat these disorders [24]. Approximately 1100 years ago, Al-Balkhí astutely distinguished between al-huzn, which refers to ordinary melancholy, and al-jaza, which denotes anxiety [5,13,25]. He further classified causes of depression into internal factors (within the body) and external factors (such as environmental or social factors) [8,20].

Al-Balkhi was among the pioneering academics who meticulously classified and delineated these diseases, recognizing them as noteworthy health concerns that necessitated meticulous diagnosis and treatment [3,7]. His approach to mental health was characterized by a profound sense of empathy and a practical mindset, in addition to recognizing symptoms; he also suggested therapeutic ways for effectively managing these problems [4,13,23,24]. Table 2 demonstrates the description and treatments of the main mental disorders proposed by Al-Balkhi.

**Table 2:** Mental disorders and treatments by Al-Balkhi This table outlines the mental disorders and treatments proposed by Al-Balkhi, adapted from various scholarly sources [4,9,13,18-20,22,24,25].

Mental disorder	Description	Proposed treatment
Anxiety (al-jaza)	Characterized by excessive worry, fear, and unease.	Implementing cognitive restructuring to modify irrational thinking, engaging in physical exercise, practicing relaxation techniques, maintaining a healthy diet, and actively participating in social activities are effective strategies for reducing anxiety.
Depression (al-huzn)	Marked by persistent sadness, loss of interest, and decreased energy.	Applying cognitive therapy to modify negative thought patterns, participate in pleasurable activities, engage in physical exercise, maintain a healthy diet, and foster social connection to elevate mood.
OCD (Al- waswasah)	Involves intrusive thoughts (obsessions) and repetitive behaviors (compulsions).	The use of cognitive-behavioral approaches involves the identification and modification of illogical beliefs, progressive exposure to situations that provoke fear, and the prevention of obsessive behaviors to limit their occurrence.
Trauma and Stress Disorders	Psychological impact of traumatic experiences leading to distress and impairment.	Psychological assistance, cognitive-behavioral approaches to processing traumatic memories, methods for managing stress, and social support to facilitate rehabilitation.
Psychosomatic Disorders	Physical symptoms are significantly influenced by psychological factors.	A comprehensive treatment that encompasses both the physical and mental components, including cognitive therapy to effectively manage stress, implementing lifestyle modifications such as dietary and activity adjustments, and employing stress reduction techniques.
Melancholia	A severe form of depression characterized by deep sadness and lack of pleasure in life.	The strategies include recognizing and questioning pessimistic thoughts, promoting engagement in enjoyable activities, engaging in physical exercise, and cultivating a supportive social atmosphere.

Al-Balkhi was a pioneering scholar who methodically identified and delineated a range of mental illnesses, including anxiety and OCD. His acknowledgment of these illnesses and their influence on overall health was revolutionary, offering early frameworks that mirror contemporary diagnostic criteria [4,19]. He stressed the significance of early diagnosis and incorporating physical and mental health treatments, foreshadowing modern holistic healthcare techniques [7,13,22].

Important contributions in psychology and psychotherapy

**Figure 2:**
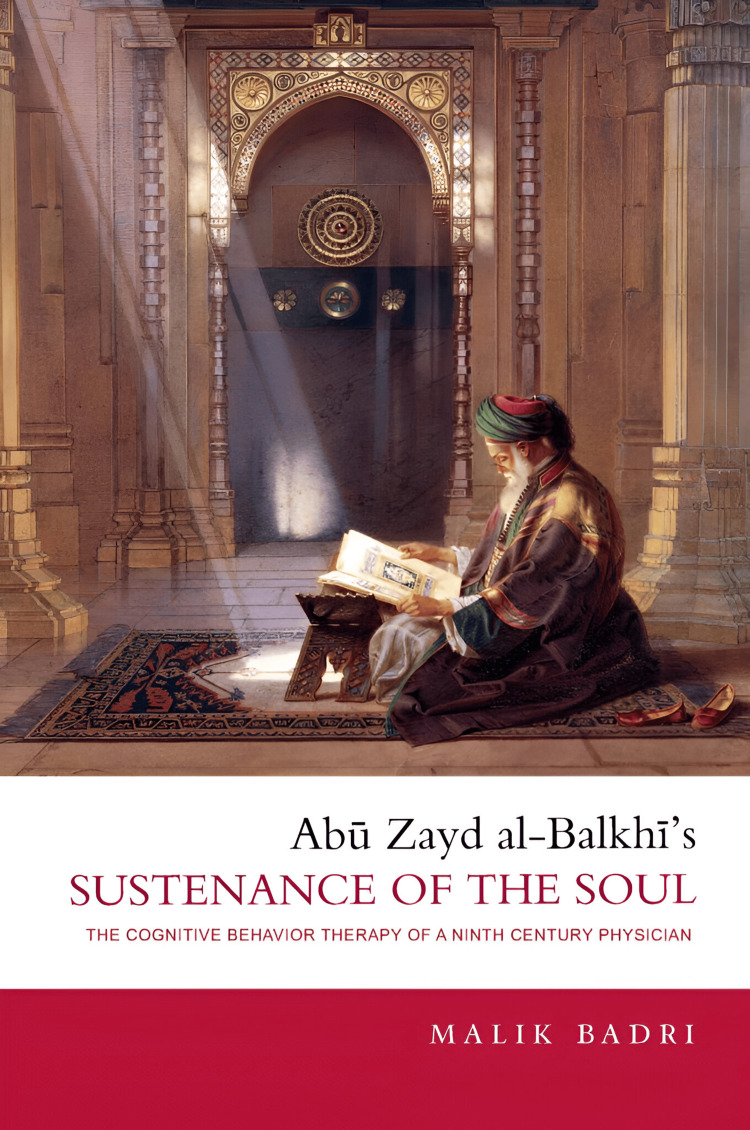
Sustenance of the Soul Book - English translated version Reproduced with permission from the International Institute of Islamic Thought [27].

Al-Balkhi revolutionized the disciplines of psychology and psychotherapy with his extensive work "Masalih al-Abdan wa al-Anfus" (Figure 2 shows the cover of a translated version of the book) [26,27]. His comprehensive approach represented a notable deviation from the medical methods of his era, which frequently addressed these areas in isolation [8,15]. In addition, Al-Balkhi's research encompassed the comprehension of the psychological effects of trauma and stress. He possessed knowledge of how external pressures and traumatic experiences could result in mental health problems, which is now acknowledged as the discipline of trauma psychology [4,8].

Al-Balkhi's achievements in the field of psychotherapy are noteworthy, specifically for his support and promotion of cognitive-behavioral techniques. His therapeutic skills were highly advanced at his time, he promoted an early version of CBT [13,24]. Al-Balkhi proposed that negative ideas and irrational beliefs play a significant role in causing emotional pain. He also stated that individuals can be taught to modify their thought patterns to enhance their mental well-being [1,4,7]. Al-Balkhi's excellent comprehension of psychological processes is shown in his tight alignment with modern CBT [2,8,26].

Al-Balkhi also underscored the significance of lifestyle and environmental factors in mental health. He acknowledged that elements such as food, physical activity, and social connections have a substantial influence on an individual's psychological well-being, based on this foundation, his prescription for managing mental health includes maintaining a healthy diet, engaging in regular exercise, and fostering strong social connections [1,13]. Al-Balkhi made significant contributions to the fields of mental health and psychology, not only through theoretical frameworks but also by implementing practical procedures. He devised activities aimed at assisting individuals in regulating their emotions, enhancing their psychological fortitude, and cultivating coping mechanisms for stress and mental disorders [2,4,7]. The purpose of these activities was to enhance cognitive resilience and foster emotional equilibrium, ideas that strongly correspond with modern psychotherapy approaches [25].

Al-Balkhi was an innovative and forward-thinking figure in the realm of mental health. For this reason, new researchers highlight the significant influence of his psychotherapy methods, specifically his early support for cognitive-behavioral techniques, establishing him as an entrepreneur in the field of mental health and psychology [1,13]. Finally, Badri, in his translation of Al-Balkhi’s book, differentiated these four main foundations of Al-balkhi: (1) A Pioneer of Psychosomatic Medicine, (2) As a Modern Counseling Psychologist, (3) The Pioneer of Cognitive Therapy, and (4) The First to Discover the Difference Between Endogenous and Reactive Depression [8].

## Conclusions

Al-Balkhi made significant and diverse contributions to psychotherapy, mental health, medicine, and geography during the Islamic Golden Age, which has had a lasting impact. His innovative contributions, particularly in the field of mental health and psychotherapy, highlight his deep comprehension of the intricate relationship between the mind and body, a notion that was far ahead of its era. Al-Balkhi's comprehensive approach to well-being, which encompassed early concepts of what is now identified as CBT, underscores his position as a forerunner to contemporary psychological methodologies. Al-Balkhi's capacity to assimilate information from various intellectual traditions, together with his pioneering advancements in the comprehension of psychosomatic medicine, establishes his position as a genuine polymath. His enduring impact on current thinking solidifies his status as a crucial figure in the annals of science and medicine.

## References

[1] Khan LA, Ganai ZA (2024). Rediscovering the comprehensive contribution of Abu Zayd al-Balkhi in the contemporary psychology. Int J Islam Psychol.

[2] Mitha K (2020). Conceptualising and addressing mental disorders amongst Muslim communities: approaches from the Islamic Golden Age. Transcult Psychiatry.

[3] Mohamed WMY (2008). Arab and Muslim contributions to modern neuroscience. IBRO Hist Neurosci.

[4] Raudah SF, Arief Y, Rahman AA (2023). Abu Zayd Al-BAlkhi’s perspective on depression and anxiety in ‘MASALIH AL-ABDAN WA AL-ANFUS’. Psikis J Psikol Islam.

[5] Shahpesandy H, Al-Kubaisy T, Mohammed-Ali R, Oladosu A, Middleton R, Saleh N (2022). A concise history of Islamic medicine: an introduction to the origins of Medicine in Islamic civilization, its impact on the evolution of global medicine, and its place in the medical world today. Int J Clin Med.

[6] Sugiarti T (2021). Analysis of the balance of the human body and spirit according to Abu Zaid al-Balkhi in cognitive psychology [Article in Indonesian]. Coution J Couns Educ.

[7] Arroisi J, Himaya NN (2023). Abu Zayd al-Balkhi’s perspective on depression countering sadness with cognitive theory in the book of mashalih Al Abdan wa al anfus. TAZKIYA J Psychol.

[8] Badri M (2013). Abu Zayd al-Balkhi’s Sustenance of the Soul; The Cognitive Behavior Therapy of a Ninth Century Physician. Gutenberg Press.

[9] Awaad R, Ali S (2015). Obsessional disorders in al-Balkhi's 9th century treatise: sustenance of the body and soul. J Affect Disord.

[10] Sadiq Z, Farwa U, Jamil MS (2022). Contribution of Muslims in the field of medical science. Res J Al-Meezan.

[11] Sugawara E, Nikaido H (2024). Abū Zayd Balḵī. Antimicrob Agents Chemother.

[12] Kamarulbahri T, Noor HM, Mat KC (2024). Integrating Islamic principles to clinical mental health care: insights from al-Balkhi’s approach to psychiatric disorder. J Sains Kesihat Malays.

[13] Awaad R, Ali S (2016). A modern conceptualization of phobia in al-Balkhi's 9th century treatise: sustenance of the body and soul. J Anxiety Disord.

[14] Mobayad T (2024). This Muslim introduced concept of mental health in 9th century. Published.

[15] Istikhari N (2021). Cognitive approach in Al-Balkhi’s mental health theory: positive psychology in the Golden Age of Islam [Article in Indonesian]. Psikologika J Pemikir dan Penelit Psikol.

[16] Ahmed JO, Kakamad KK, Najmadden ZB, Saeed SI (2024). Abu Bakr Muhammad Ibn Zakariya Al-Razi (Rhazes) (865-925): the founder of the first psychiatric ward. Cureus.

[17] Awaad R, Ali S (2023). The original self-help book: Al-Balkhi’s 9th century “sustenance of the body and soul”. Spiritual Clin Pract.

[18] Ozkan-Rashed Z (2023). The manuscript of al-Balḫī’s Masạ̄liḥ (al-abdān) wa-l-anfus according to Fuat Sezgin and its present evaluation. The 2nd International Prof. Dr. Fuat Sezgin Symposium on History of Science in Islam Proceedings Book.

[19] Sarhan W (2018). The contribution of Arab Islamic civilization to mental health. Arab J Psychiatry.

[20] Haque A (2004). Psychology from Islamic perspective: contributions of early Muslim scholars and challenges to contemporary Muslim psychologists. J Relig Health.

[21] Ansari S, Iqbal N (2023). Contributions of Muslim medieval scholars to psychology. Arch Psychol Relig.

[22] Musfihin M (2019). Body and Soul balance from Abu Zaid Al-Balkhi’s perspective. J Stud Insa.

[23] Awaad R, Mohammad A, Elzamzamy K, Fereydooni S, Gamar M (2019). Mental health in the Islamic Golden era: the historical roots of Modern Psychiatry. Islamophobia and Psychiatry: Recognition, Prevention, and Treatment.

[24] Söylev OF (2020). Obsessive-compulsive disorders from the perspective of religion: modern approaches and the contributions of Abū Zayd al-Balkhī. Cumhur Ilah Derg.

[25] Taibah SA, Hawadi LF, Al-Asyhar T (2023). Psychotherapy: a comparison of Abu Zayd Al-Balkhi and CBT. Psikis J Psikol Islam.

[26] Cucchi A (2022). Integrating cognitive behavioural and Islamic principles in psychology and psychotherapy: a narrative review. J Relig Health.

[27] Al-Balkhi AZ. Abu Zayd Al-Balkhi’s Sustenance of the Soul (2024). Abu Zayd Al-Balkhi’s sustenance of the soul: the cognitive behavior therapy of a ninth century physician. https://iiit.org/en/book/abu-zayd-al-balkhis-sustenance-of-the-soul/.

